# Photofunctionalization of Titanium: An Alternative Explanation of Its Chemical-Physical Mechanism

**DOI:** 10.1371/journal.pone.0157481

**Published:** 2016-06-16

**Authors:** Marco Roy, Alfonso Pompella, Jerzy Kubacki, Jacek Szade, Robert A. Roy, Wieslaw Hedzelek

**Affiliations:** 1 Prosthodontic Department, Poznan University of Medical Science, Poznan, Poland; 2 Department of Translational Research and New Technologies in Medicine and Surgery, University of Pisa Medical School, Pisa, Italy; 3 A. Chelkowski Institute of Physics, University of Silesia, Katowice, Poland; 4 Silesian Center for Education and Interdisciplinary Research, Chorzów, Poland; 5 Private Practice, Pisa, Italy; VIT University, INDIA

## Abstract

**Objectives:**

To demonstrate that titanium implant surfaces as little as 4 weeks from production are contaminated by atmospheric hydrocarbons. This phenomenon, also known as biological ageing can be reversed by UVC irradiation technically known as photofunctionalization. To propose a new model from our experimental evidence to explain how the changes in chemical structure of the surface will affect the adsorption of amino acids on the titanium surface enhancing osteointegration.

**Methods:**

In our study XPS and AES were used to analyze the effects of UVC irradiation (photofunctionalization) in reversing biological ageing of titanium. SEM was used to analyze any possible effects on the topography of the surface.

**Results:**

UVC irradiation was able to reverse biological ageing of titanium by greatly reducing the amount of carbon contamination present on the implant surface by up to 4 times, while the topography of the surface was not affected. UVC photon energy reduces surface H_2_O and increases TiOH with many –OH groups being produced. These groups explain the super-hydrophilic effect from photofunctionalization when these groups come into contact with water.

**Significance:**

Photofunctionalization has proven to be a valid method to reduce the amount of hydrocarbon contamination on titanium dental implants and improve biological results. The chemisorption mechanisms of amino acids, in our study, are dictated by the chemical structure and electric state present on the surface, but only in the presence of an also favourable geometrical composition at the atomical level.

## 1. Introduction

Titanium is a material widely used in the medical field for orthopedic prosthesis and implant dentistry. A lot of time and effort has been put into analysing the chemical and physical nature of the oxide layer on its surface [1]. It is well known to be a non-toxic, stable, easily available and to have great mechanical properties that are low modules of elasticity, a high strength-to-weight ratio and most of all an excellent resistance to corrosion [2, 3]. Nevertheless, it still seems possible to make new discoveries that enhance its qualities [4]. Freshly cut titanium seems to be more active in promoting protein attachment and cell adhesion than titanium aged 4 weeks or more [5, 6]. The decrease in biological activity is related to the inevitable hydrocarbon deposition on its surface also known as biological ageing [6]. To counteract this phenomenon removal of the contamination present on the surface is necessary; this will in turn restore and even enhance the properties of titanium. UVC Photofunctionalization has been introduced as a valid method to remove the hydrocarbon contamination [7]. UV, ultraviolet radiation, corresponds to electromagnetic radiation shorter than visible light, in the wavelength range of 100–400 nm. Our study was focused on wavelengths in the 100–280 nm interval, corresponding to UVC rays. The recontamination of the surface is unfortunately a very fast phenomena considering that it takes only 4 weeks afor the freshly cut or previously photofunctionalized titanium implants to be covered with hydrocarbons no matter the type of surface treatment they have undergone [8]. The aim of this study was to analyze the characteristics of dental implants photofunctionalized following the Ushio Therabeam^®^ SuperOsseo protocol. Emphasis was put in calculating the concentration of the various elements on the surface by comparing AES (Auger Electron Spectroscopy) with XPS (X-ray Photoemission Spectroscopy), being AES able to analyze a more superficial layer than the XPS. Moreover, we would like to propose a new model to explain how photofunctionalization modifies the titanium surface chemically and how this change could affect the biological properties, which in turn will enhance the process of subsequent osteointegration.

## 2. Materials and Methods

### 2.1. Implants

Commercially available Osteoplant Base^™^ and Rapid^™^ titanium dental implants were used in our experiments. Geometrically the Base implant is cylindrical and the Rapid being instead conical. The implant surface was treated by the factory, sand-blasted or acid etched according to their protocols, to increase surface roughness and therefore the extension of available contact area between bone and the dental implant. The implants were studied as received from the company and immediately after 12 min of UVC irradiation treatment with Ushio TheraBeam^®^ SuperOsseo apparatus.

### 2.2. AES, SEM and XPS analysis

The implant surfaces, versions (RAPID and BASE) were studied by SEM (Scanning Electron Microscope), AES (Auger Electron Spectroscopy) and XPS (X-ray Photoelectron Spectroscopy) methods, in order to analyse the changes of surface morphology and chemistry. Measurements were performed with PHI5700/660 Physical Electronics spectrometer. SEM images were obtained at 10kV acceleration voltages and approximately ~20nA primary beam current. AES spectra were recorded in integral mode. Chemical distribution maps were obtained for carbon, titanium and oxygen. Photoelectron spectra were obtained with the use of a monochromatic Al anode x-ray source with energy hν = 1486.8 eV of radiation K_α_. Survey spectra and core lines of Ti2p, O1s, C1s, N1s and F1s were recorded. The Shirley type of background was subtracted before analysis. The fitting procedure was applied to the analysis of a shape of core lines by Seampik software. Moreover, atomic concentration of the elements was calculated from the auger and photoemission spectra by using the Multipak (Version 9.6.0.15) software. The calculation of atomic concentrations with XPS and AES was based on relative peak intensities, modified using relative sensitivity factors (RSFs). Atomic concentration for each element was determined using the equation:

$$\text{Atom}\;\%\;\text{of}\;\text{element}\;\text{X}\;=\;[({\text{I}}_{\text{x}}/{\text{S}}_{\text{x}})\;/\;(\sum {\text{I}}_{\text{i}}/{\text{S}}_{\text{i}})]\;\text{x}\;100$$

where I_x_ is intensity or peak area and S_x_ is the relative sensitivity factor for the element. Critical information including transmission function parameters and instrument configuration were stored with PHI XPS data files. Empirically derived relative sensitivity factors (ERSFs) and calculated average matrix relative sensitivity factors (AMRSFs) were both available in MultiPak for AES data reduction. More information can be found in the work by Seah [9].

## 3. Results

### 3.1. Surface analysis

**Fig 1:**
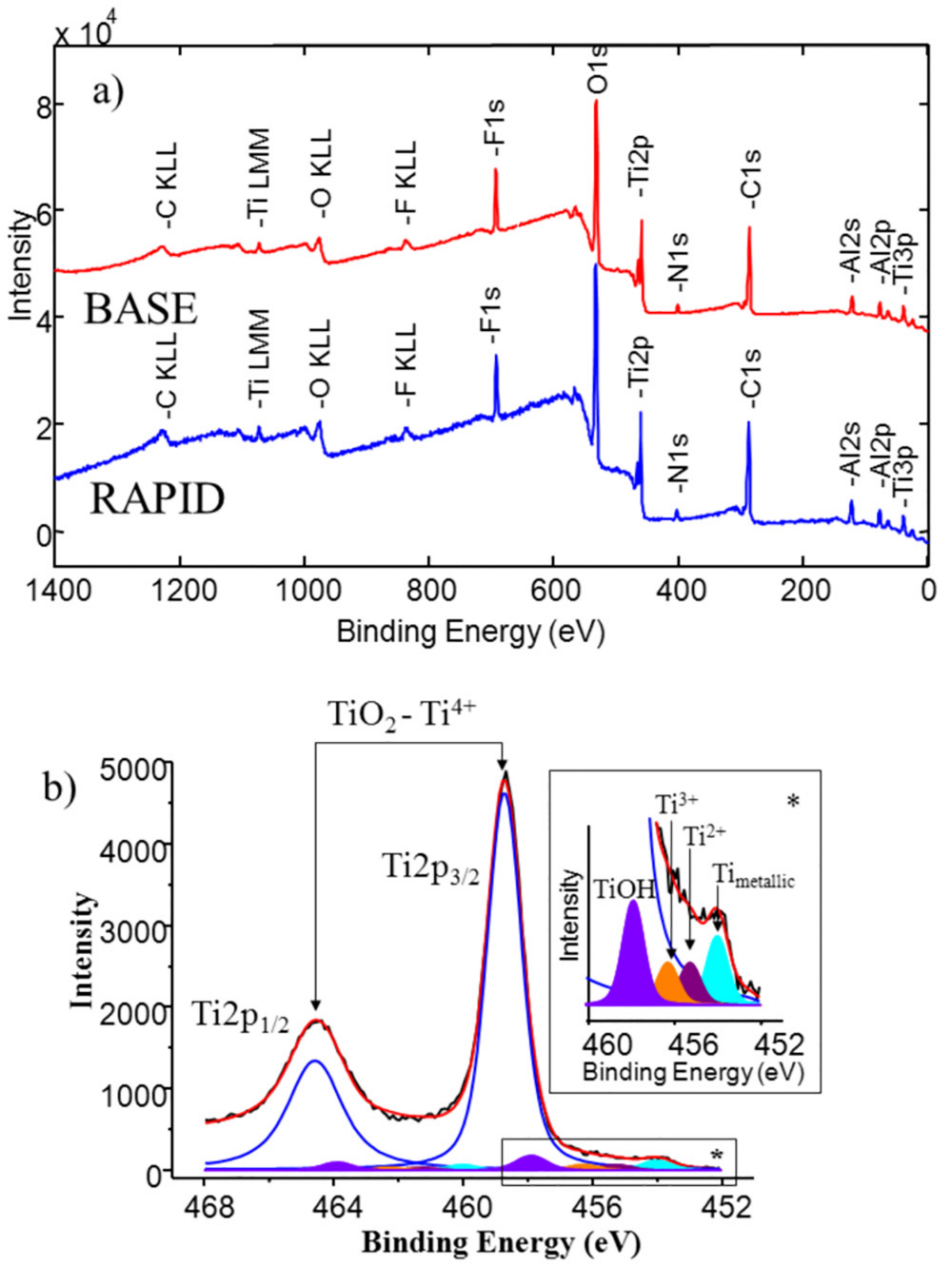
A) The XPS survey spectra obtained for the BASE(red) and RAPID (blue) implants as received. On both surfaces Ti, O, C, Al and F were detectable. B) High resolution XPS spectrum of the Ti2p core line for RAPID implant. The shape of the 2p doublet was fitted by to five sub-doublets. The doublet with the highest intensity corresponded to TiO_2_ component. The enlarged region presented the Ti2p_3/2_ peaks corresponded to hydrated water Ti-OH, various oxidation states and metallic state of titanium.

In Fig 1a the XPS survey spectra of the reference BASE and RAPID implants are shown. The elements Ti, O, C, Al and F were detected. Titanium, oxygen and carbon were the main elements present on the implant surface, while aluminium and fluoride were deposited during the sand-blasting and acid etching procedures during manufacturing.

Table 1 shows atomic concentrations, obtained by XPS measurements, on the surface of both implant types. We found titanium (about 7% and 6% for the BASE and RAPID samples, respectively), oxygen (about 37%), aluminium (about 8%) and nitrogen (about 2%). The level of carbon contamination was about 39%.

**Table 1:** Atomic concentration of carbon, nitrogen, oxygen and titanium calculated from C1s, N1s, O1s, Al2p, Ti2p and F1s core lines for BASE and RAPID implants.

	Carbon	Nitrogen	Oxygen	Aluminum	Titanium	Fluoride
**BASE**	39%	2%	37%	8%	7%	7%
**RAPID**	39%	2%	38%	8%	6%	7%

The shape of the Ti2p doublet presented in Fig 1b consists of several components which can be ascribed to presence of Ti-OH bonds and various titanium oxides as reported by Kang [10]. The binding energy of the main peak of Ti2p_3/2_ state at E = 458.7 eV confirmed the presence of TiO_2_ on the implant surface [11]. The metallic form of the titanium was also detected on the surface reference materials. We focused our study on the analysis of Ti, O and C.

**Fig 2:**
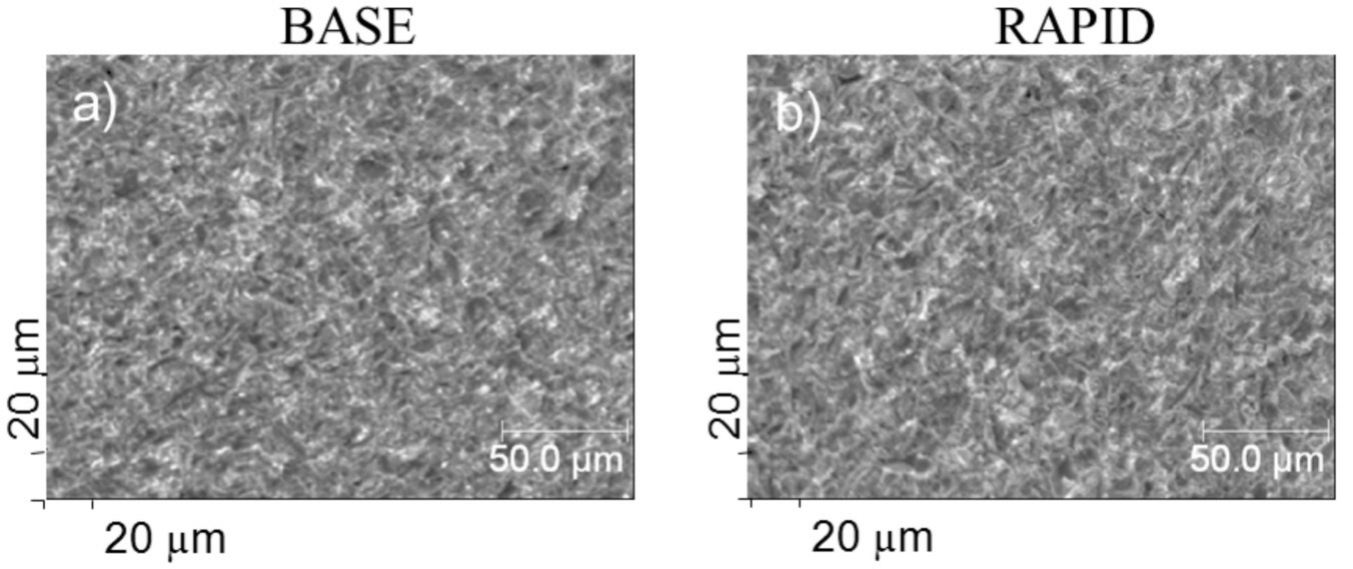
Electron microscope images obtained from magnification x500 recorded for BASE (a) and RAPID (b) implants as received. The contrast from light to dark areas suggest a considerable degree of roughness of the analysed areas. Comparing (a and b) the surfaces of both type implants are quite similar.

In Fig 2a and 2b the SEM images of the reference BASE and RAPID implants are shown. The distribution of different contrast from light to dark areas indicates a considerable degree of roughness of the analysed areas. The RMS value obtained from the AFM scan (Solver P47 NT-MDT instrument, non-contact mode) with size 15 x 15 μm was 0.4 μm. However, the surfaces of both type implants are quite similar, as can be expected considering that both were sandblasted and acid-etched during manufacturing.

**Fig 3:**
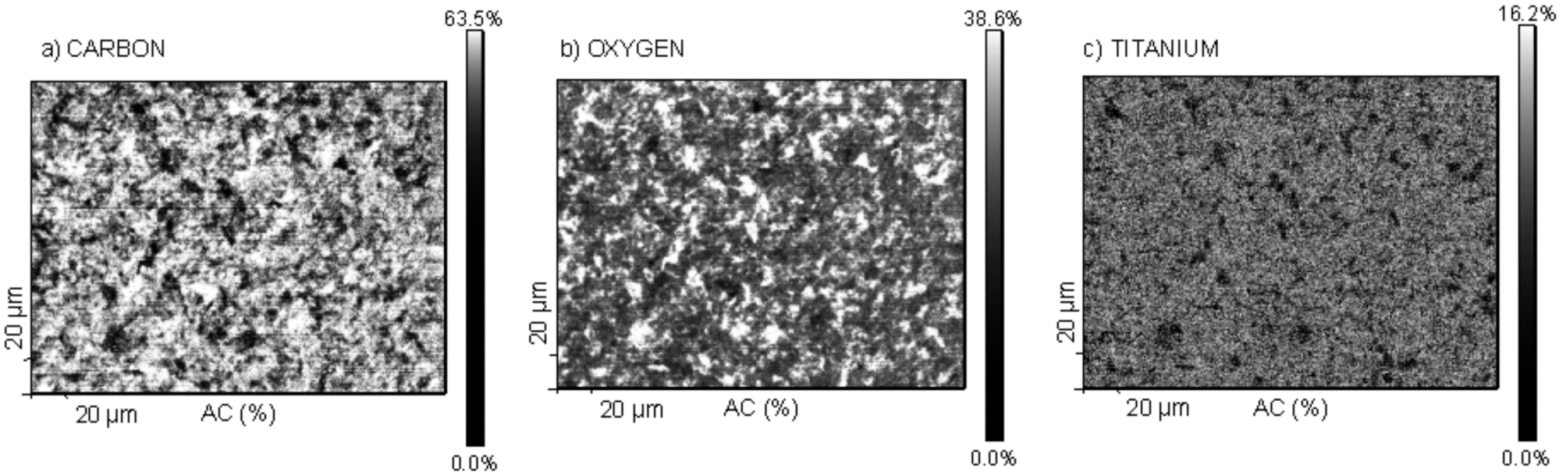
The chemical distribution maps of oxygen (a), carbon (b) and titanium (c) obtained for the RAPID implant. The distribution of the elements is grain type with carbon spread all over the surface while titanium is almost homogenous.

The chemical distribution of carbon, titanium and oxygen has been shown in Fig 3a, 3b and 3c and presents a non-homogeneous grain type distribution for these elements on both implants. The carbon is spread all over the analysed area. Its atomic concentration in some parts achieves 63%. The oxygen is also very prominent on the surface. However, its maximum concentration of 38,6% is achieved only in a confined area. Distribution of titanium with respect to carbon and oxygen is almost homogeneous. However, its value of atomic concentration is very low. The presented results confirm the hypothesis that the implant surface is covered mainly by carbon and oxygen compounds.

The present data shows the typical behaviour of titanium after biological ageing. The surface of freshly cut titanium is rapidly oxidized and consists of rows of bridging oxygen ions with 5 fold Ti4+ ions (Ti5c), in plane oxygen, fully coordinate titanium (Ti6c) and O-vacancies defined as defects. This composition can be stable only if kept in the UHV (Ultra High Vacuum), if exposed to the atmosphere the unsaturated Ti5c atoms form bonds with H_2_O molecules forming therefore, hydroxyl groups (-OH). This coverage is the reason for the hydrophilic character of fresh titanium, however the surface still is not completely stable and continues to form bonds also with hydrocarbons, explaining why there is a high presence of both oxygen and carbon on implants which are older than five weeks.

### 3.2. XPS analysis after UVC irradiation

**Fig 4:**
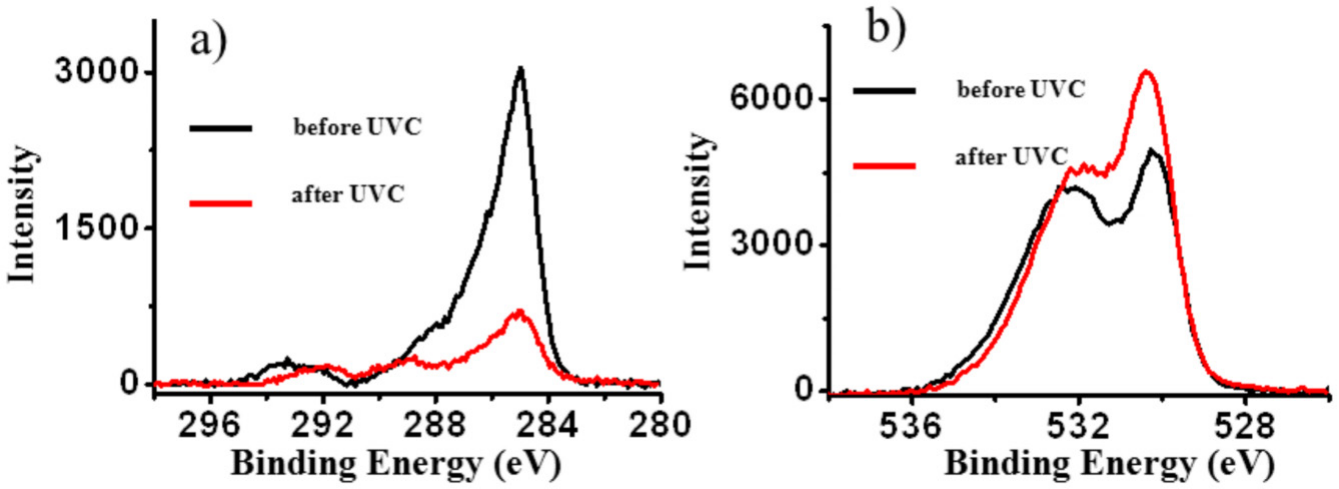
The C1s (a) and O1s (b) core lines recorded for as received and after photofunctionalization samples. The lines in red show the decontamination effect of the UVC irradiation decreasing the hydrocarbons peak and increasing the Oxygen peak.

In order to distinguish the type of carbon and oxygen contamination and to verify the effectiveness of UVC in decontaminating titanium surfaces, the core lines O1s and C1s were recorded for the RAPID and BASE implants before and after treatment. Because obtained results are the same no further results for the RAPID material will be presented. In Fig 4a and 4b we present results of C1s and O1s measurements obtained for the implant as received and after photofunctionalization.

**Fig 5:**
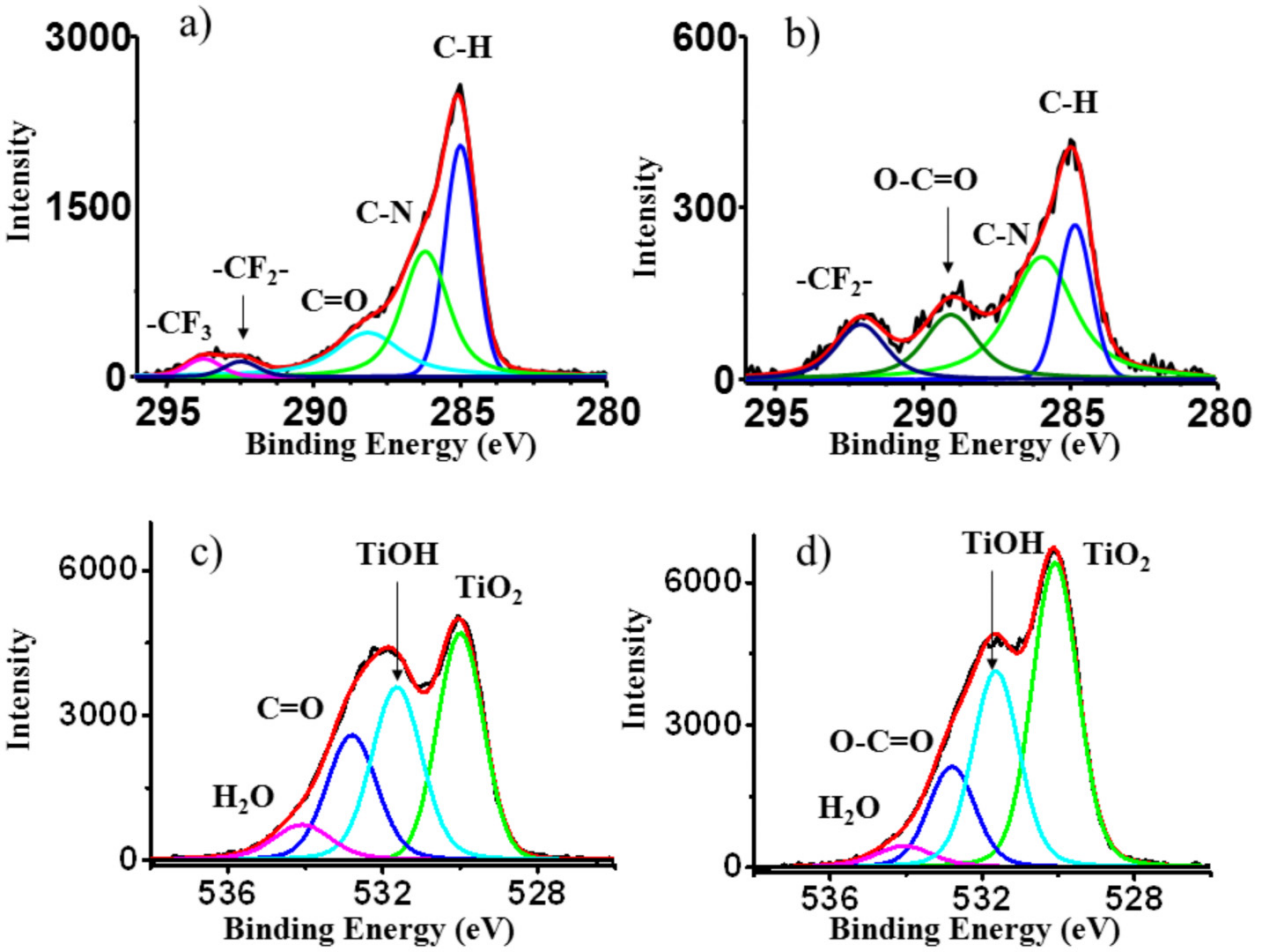
Line shape analysis of C1s and O1s spectra for the implant as received (a and c) and after photofunctionalization (b and d). Comparing (a and b) the intensity of the peak at 285 eV corresponding to the carbon contamination is highly reduced. The oxygen lines (c and d) show an increase in oxygen peak.

The significant decrease of carbon C1s signal after the USHIO UVC irradiation treatment procedure was clearly visible, while in the case of oxygen only a small increase in the signal was observed. Additionally, the shape of both measured lines is combined with additional components. The results of the deconvolution of the experimental lines is presented in Fig 5a–5d.

The C1s spectral profile in Fig 5a consists of five components. The component with the highest intensity at energy E = 285 eV corresponds to the C of hydrocarbons. The second component corresponds to the C-N chemical group, and the third to the CO group of molecules present on the surface. The last two components with the highest binding energy can be ascribed to the presence of—CF_3_ and—CF_2_ groups. The analysis of the O1s core lines is reported in Fig 5c. The O1s line shape consists of four components. The component with the highest intensity corresponds to TiO_2_, the second corresponds to TiOH groups, the next component can be ascribed to C = O groups and the last one corresponds to H_2_O linked to surface Ti atoms. The changes in intensity of C1s and O1s spectra after UVC irradiation are reported in Fig 5b and 5d, respectively. The intensity of all four components for carbon, corresponding to C-H, C-N, O-C = O and—CF_2_- bonding, drastically decreased. In the case of the O1s spectrum the signal corresponding to oxygen atoms of TiO_2_ slightly increased. The peak at 534 eV corresponds to H_2_O molecules present on TiO2. Comparing its intensity before and after UVC irradiation a slight decrease was observed. On the other hand, a minor increase in the intensity of TiOH was observed while, that of carbon dioxide was slightly decreased.

The results of atomic concentration calculations for C, O and Ti obtained by XPS and AES methods before and after photofunctionalization are presented in Table 2. In the case of N only the XPS value is given, because in AES the peak corresponding to nitrogen (N KLL) overlaps the peak of Titanium (LMM), therefore the N component is impossible to distinguish. Hence, the Ti LMV Auger peak was used for AES calculation of atomic concentration. The values of the atomic concentrations calculated for the RAPID implant as received from the AES and XPS data were almost the same, confirming the equivalence of the two methods. The atomic concentration of C decreased by about four times, while in the case of O and Ti an increase of atomic concentration was observed. The amount of N decreased by about 1%.

**Table 2:** Atomic concentration calculations obtained from the AES and XPS spectra for the surfaces of RAPID implant as received and after UVC irradiation.

	Carbon	Nitrogen	Oxygen	Titanium
**AES/XPS *taken out of the box***	47/43	XPS—2	45/47	8/8
**AES/XPS *UVC 12 min cycle***	13/14	XPS—1	75/69	11/13

## 4. Discussion

In our study we used 2 different types of implants with a different macroscopic geometry to prove that biological ageing is not selective and that photofunctionalization decontaminates the surfaces independently from the geometry of the implant. On both of the control RAPID and BASE implants the amount of carbon deposition on the surface is almost identical as seen by XPS and AES analysis (Table 1). The peak recorded at 285 eV in the XPS spectrum corresponds to the presence of different C containing molecules on the surfaces. After UVC photofunctionalization the amount of C present on the surface decreased 4 times. Moreover, the intensity of O and Ti peaks increased suggesting that an easier ionization of O and Ti atoms occurred on a less contaminated surface. By XPS analysis we observed that photofunctionalization was also able to decrease the contamination revealed by the CF2 component of the C1s spectrum, originating from the acid-etching procedure during manufacturing.

On TiO2 the peak at 534 eV corresponds to H_2_O molecules [12], which inevitably attach on its surface when exposed to the atmosphere oxidizing Ti^3+^ to Ti^4+^ [13]. UVC irradiation was also able to decrease the amount of H_2_O and increase the amount of TiOH. UVC photon energy induces a one-electron oxidation with water to produce a hydroxyl radical OH and dissociation of H^+^ [14]. These hydroxyl groups together with the O-Vacancies, present on an almost C free surface are able to form more OH^-^ groups which explains the super-hydrophilic effect from photofunctionalization when these groups come into contact with water. Highly hydrophilic surfaces seem to improve their interactions with biological fluids, cells and tissues [4, 15].

Hayashi et al. have described the importance of a low C/Ti ratio in order to increase osteoblastic activity [16]. In our study this ratio after photofunctionalization was reduced by almost 4 times. Photofunctionalization has been hypothesized to increase the attachment of cells to the implant surface thanks to the change in the electrical charge of the surface [5, 17]. In the next section we would like to introduce an alternative model which could also explain the better affinity of cells for the titanium surface.

Some researchers have proposed that the exposure of TiO_2_ to UV results in an excitement of an electron from valence band to conduction band, leaving the TiO_2_ surface electropositive [18]. The excitation process is followed by a very fast de-excitation so that this charging phase is not stable enough to give any macroscopic effect. This mechanism is instead exploited in photovoltaic cells, as described by Castellotte et al. [14] where the excited electrons are removed by electrochemical reactions and charge carriers that are missing missing over a simple TiO_2_ surface. Before introducing our model, we would like to point out that TiO_2_ always covers with a thickness of few microns on any TiO_2_ surface exposed to water, and that this is the real interface with environment for a Ti dental implant. TiO_2_ can expose different faces and in practice a mix of all of them will be present on the surface of the implant. In the bulk of a TiO_2_ crystal each Ti atom is surrounded (coordinated) by 6 atoms, while on the surface different coordinations are possible. Our major interest is in the penta-coordinated form also known as Ti5c where the O atom “on the top” is removed from the exposed surface. This particular form of Ti atom plays a major role in surface protein attachment due to its tendency to form chemical bonds with the O and S atoms present in biological molecules, and giving the surface its charge. When freshly cut Ti exposes both the fully coordinated Titanium (Ti6c) and the penta-coordinated Ti5c, which is lacking a bond to completely saturated, it becomes more reactive. The concept of biological ageing can be explained as the saturation of the Ti5c sites with the O of the carboxyl groups present on hydrocarbon chains. In our model UVC high energy photons are able to chemically break the relatively weak bonds between the carboxyl group of the chemisorbed compound and the surface Ti5c, hence giving the chance for Ti5c to make a bond with the O, N and S atoms present on proteins and therefore enhance the attachment of cells, and in turn the osteointegration process. This process has already been presented for the adsorption of monocarboxylic acids on TiO_2_ [19] and is also used to explain amino acid adsorption on TiO_2_ nanoparticles [20].

Our interest is in the adsorption of amino acids, as they are the units which compose the proteins present in tissues and on cells. Amino acids present one end with an acidic carboxylic acid and the other one with a basic amine group. Tonner and co-workers described proline and glycine adsorption on TiO_2_ and concluded that it occurs through the carboxyl group, with proton transfer to the surface taking place [21]. They also showed hydrogen bonding *via* the amine group towards the surface O atoms, which led to further stabilization of the adsorption of amino acids to the surface [22, 23]. The highest adsorption energy was obtained for a model where the carbonyl oxygen of glycine bonds to a Ti5c site. In this model, the hydroxyl group forms a hydrogen bond to a twofold coordinated oxygen atom, and the amine group is bonded to Ti5c *via* the nitrogen lone pair. However, the authors have shown that the energy difference between this model and one involving adsorption *via* the deprotonated carboxyl group—or indeed solely *via* the amine group—is very small, thus it is possible that all three modes of adsorption may be present [22]. It was proven again by Flemming and co-workers that proline is adsorbed in a bi-dentate geometry *via* the carboxyl group [22]. Moreover, glycine adsorption was seen to occur preferentially *via* deprotonation [2] of the carboxyl group as seen for acetic and formic acid. We would also like to point out that the difference in charge is not enough to produce the chemical bond between amino acids and Ti5c. It has been proposed by Li et al. [24] that the amino acid cysteine, which besides the carboxyl group has an SH group, can readily attach to the TiO_2_ surface preferably *via* the carboxyl group. It has been observed that for rutile titanium, one form of TiO_2_, that this adsorption mode is favoured by the geometry of the surface, as the distance between two Ti5c atoms fits very well the distance between the O atoms of a carboxyl group, while, due to its larger radius a sulphur atom cannot easily approach a Ti5c atom of the rutile surface. Therefore, we suggest that the photofunctionalization model should not focus just on a charge interaction picture but on a more complex chemisorption mechanism that is well established in surface science for amino acids on TiO_2_ surfaces. The adsorption of amino acids on carbon free titanium surfaces has been widely analyzed through theoretical models and confirmed with experimental studies [23–32].

**Fig 6:**
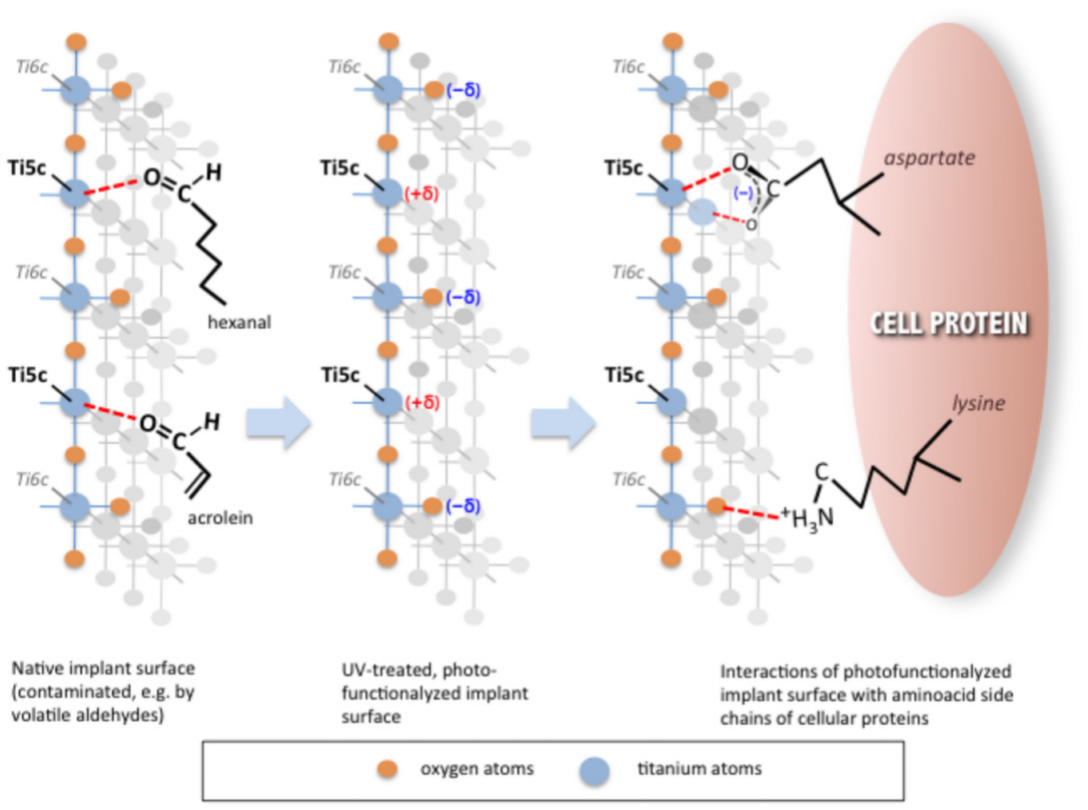
Scheme representing the interactions of carboxyl and amine groups with TiO2 surface when exposed to the atmosphere. The surface shown is TiO2 (110), with Ti (light blue) and O (orange). See text for details.

In Fig 6 we propose our model, summarizing the effect the chemio-adsorption process of hydrocarbons on TiO2 ("biological ageing"), and the role of photofunctionalization as far as cleaning and enhancing the TiO_2_ surface. When exposed to atmosphere, TiO_2_ surface can bind pollutant hydrocarbons through interactions with carboxyl and amine groups. The surface shown in the Figure is TiO_2_ (110), just as an example of the different faces present in the polycristalline oxide forming on the implant surface. Carboxyl and amine groups are taken into consideration since they are typical grouping exhibited by amino acid side chains in protein structure. The left panel shows the interaction between C = O groups typical of gaseous contaminants present in the atmosphere (e.g. volatile aldehydes) with the penta-coordinated Ti exposed on the implant surface. The dotted line indicates that the interaction (chemi-adsorption) is one order of magnitude weaker than the typical bond energy in organic molecules. The prolonged exposure to UV photons—much higher in energy as compared to visible light—is able to break such weak “bonds”, thus removing the contaminants from the surface and re-establishing a clean surface (central panel). The scheme also shows the partial charges present on the surface (+/–delta), due to the difference in electronegativity of Ti as compared to O atoms. The deprotonated carboxyl groups (COO^-^) and protonated amine groups (NH_3_^+^) typically present in aqueous environment can easily interact with such partial charges present on the photofunctionalized surface, thus enhancing the anchorage of proteins (right panel). The bi-dentate interaction between COO^-^ and two contiguous Ti atoms has been proven very effective from this point of view [21–31].

In clinical practice the possibility of achieving a direct bond between titanium and amino acids can have a great impact in terms of attachment strength of the cells to the surface. As a result of this process osteointegration would be enhanced providing a more stable implant, and would improve the long term survival rate of the implants. Especially beneficial could be its use in cases of poor bone quality. In addition, it can be expected that the exposure of all implants to the same type of photofunctionalization procedure before use will create the same starting conditions in all patients, thus allowing the clinician to make more accurate comparisons of the individual outcomes.

## 5. Conclusions

Photofunctionalization (UVC) has proven to be a valid method to reduce the amount of.

The results of our investigations allow to conclude that UVC photofunctionalization are a valid procedure to remove hydrocarbon contamination from titanium dental implants. In the model we are proposing, UV-induced changes in chemical structure of the surface can explain the positive biological effects reported in literature. The energy carried by UV rays is able to break the bonds between Ti5c atoms and the O and/or N atoms of contaminant molecules. Chemically active Ti5c sites then become available for the attachment to O, N, S atoms present in any protein, leading to improved biocompatibility of the implant. However, the chemisorption process between amino acids and titanium atoms on the surface is dependent not only on the surface charge present and electronegativity of the interacting atoms, but also on the atomic geometry of both the implant surface and the adsorbed molecule.
